# Hearing: PCBs Alter Brain Development

**DOI:** 10.1289/ehp.115-a400

**Published:** 2007-08

**Authors:** Adrian Burton

Exposure to the non-coplanar polychlorinated biphenyl PCB95 during gestation and nursing causes abnormal development of the auditory cortex in rats, affecting the brain’s representation of what is heard, according to new research in the 1 May 2007 issue of *Proceedings of the National Academy of Sciences*. Since children with autism and other developmental disorders show abnormal responses to sound, suspicions have been raised that PCB95 and similar molecules in the environment might promote this problem and perhaps other language/cognition disorders in children.

Prior to their being banned in the late 1970s as potential carcinogens, PCBs became ubiquitous environmental pollutants that continue to threaten human health. Their molecular stability has maintained them intact, and they have entered the food chain, accumulating in the fat of exposed organisms.

Most of the early work on PCB-associated health problems focused on the coplanar molecules, but there is now evidence that non-coplanar PCBs may cause trouble of their own (coplanar and non-coplanar refer to the chemical structure of the PCBs in question). “They are reported to prevent dopamine production in monkey brains, to alter behavior in rats, and may even alter neuropsychological functioning in children,” explains first author Tal Kenet, a faculty member at Harvard Medical School and Massachusetts General Hospital. “Our research suggests they cause abnormalities in the development of the auditory part of the rat brain.”

The researchers fed pregnant rats 6 mg/kg of PCB95 in corn oil daily from day 5 of pregnancy until the weaning of their pups. “We then mapped the boundary and response characteristics of the primary auditory cortex of the pups using a series of electrodes implanted in the brain,” says Kenet. “Individual neurons were monitored to see which characteristic sound frequency they responded to.” The auditory cortex is one of the first sensory systems to mature.

The maps of the PCB95-exposed rats were found to be oddly shaped and had “holes” in them where neurons seemed not to respond to sound. The maps also included many neurons that showed a lack of frequency selectivity, and the typical posterior-to-anterior distribution of neurons responding to ever higher frequencies was disorganized.

“This must affect how their brains interpret sound,” says Kenet. “In addition, we recorded notable imbalances in inhibitory and excitatory signaling between the auditory cortex nerve cells. Without proper balancing, the correct representation of sound cannot be guaranteed. Importantly, children with autism show evidence of imbalances between excitation and inhibition in the brain, but whether it’s the same type of imbalance remains to be explored.”

The researchers also found the plasticity of the PCB-exposed cortices to be abnormal. Usually, if rat pups are exposed to a particular tone, the area of the cortex that deals with that frequency expands. “That did not happen in the PCB-exposed pups,” says Kenet.

“Epidemiological studies have found that children with prenatal PCB exposure do more poorly on tests of verbal learning and memory,” says Susan Schantz, a professor of veterinary biosciences at the University of Illinois at Urbana–Champaign College of Veterinary Medicine. “These exciting new findings suggest that underlying changes in the development or plasticity of the auditory cortex may be responsible for those effects.” However, Schantz cautions that the rats in these studies received a very high dose of a very potent PCB congener. “In my opinion,” she says, “it is unlikely that human infants, even those living in highly polluted areas, would be exposed to similar concentrations. We also need to keep in mind that any effects observed in humans are likely to be much more subtle than the striking changes observed in these rats.”

“It would be interesting to know whether animals closer to humans develop disorders resembling autism or other cognitive problems after [environmentally relevant] PCB95 exposures,” remarks Jesús Pastor, a senior researcher at the Centre for Environmental Sciences in Madrid, Spain. “That might help reveal how serious this problem could be.”

Since PCBs can be passed on to human infants in breast milk, the report raises the question of whether some mothers in highly polluted areas—perhaps those whose family history points toward a possible genetic risk of autism spectrum disorders—should bottle-feed rather than breastfeed. However, “some research shows that breast-feeding may actually lessen the negative impact of prenatal exposure, even though children who are breastfed have higher overall body burdens of PCBs,” says Schantz.

## Figures and Tables

**Figure:**
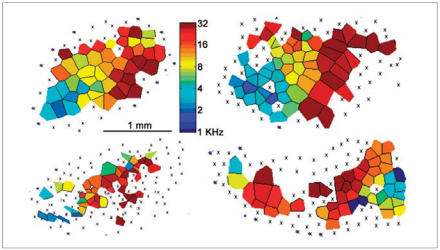
Top left shows a tonotopic map of the primary auditory cortex of a normal rat pup. At the left end of the map are neurons that are selective for low-frequency tones (blues); at the other end are neurons that respond only to high-frequency tones (reds). This pattern is usually smooth (i.e., no holes), continuous (gradually changing from one end to the other), and elliptical in shape. The other three examples above are from rats exposed to PCB95. These maps are neither continuous nor smooth, are very disorganized, and have erratic shapes. **Source:** Kenet T et al. 2007. Perinatal exposure to a noncoplanar polychlorinated biphenyl alters tonotopy, receptive fields, and plasticity in rat primary auditory cortex. Proc Natl Acad Sci USA 104(18):7646–7651.

